# Supplementary material for: In-vivo and in-vitro toxicity evaluation of 2,3-dimethylquinoxaline: An antimicrobial found in a traditional herbal medicine
Source: PLoS One. 2024 Aug 20;19(8):e0300079. doi: 10.1371/journal.pone.0300079 (PMC11335103; doi:10.1371/journal.pone.0300079)
Supplement: Attachment — Submitted filename: response letter toxicity.docx [file pone.0300079.s002.docx]

3. To comply with PLOS ONE submissions requirements, in your Methods section, please provide additional information regarding the experiments involving animals and ensure you have included details on (1) methods of sacrifice, (2) methods of anesthesia and/or analgesia, and (3) efforts to alleviate suffering.

In this study, specific criteria have been employed to evaluate the condition of animals and determine whether euthanasia is warranted. These criteria encompass a range of factors, including the animal's overall health status, behavior, pain levels, distress, and response to treatment or interventions. Additionally, various physiological parameters are monitored, such as body weight, body temperature, respiratory rate, heart rate, and clinical signs of disease or suffering.

It is imperative that decisions regarding euthanasia adhere to established guidelines and ethical principles to minimize animal suffering and ensure humane treatment. These guidelines are typically delineated in institutional animal care and use protocols, which are formulated based on regulatory requirements, scientific norms, and ethical considerations.

This study operates in accordance with the ethical guidelines and research protocols established by the Research Ethics Committee (Faculty of Pharmacy, KAU, Jeddah, SA, Reference No. PH-1443-17).

We note that the grant information you provided in the ‘Funding Information’ and ‘Financial Disclosure’ sections do not match.

When you resubmit, please ensure that you provide the correct grant numbers for the awards you received for your study in the ‘Funding Information’ section.

Thank you for bringing this to our attention. We apologize for the discrepancy in the grant information provided. We will ensure that the correct grant numbers are included in the 'Funding Information' section upon resubmission.

The corrected Grant number is: IFPIP:1281-140-1443

Thank you for stating the following financial disclosure:

“Grant number IFPIP:1199-140-1443- 260449 from the Instructional Improvement Fund supported this study.  Saudi Arabian Moe and King Abdulaziz University, DSR in Jeddah.”

Please state what role the funders took in the study.  If the funders had no role, please state: "The funders had no role in study design, data collection and analysis, decision to publish, or preparation of the manuscript."

If this statement is not correct you must amend it as needed.

Please include this amended Role of Funder statement in your cover letter; we will change the online submission form on your behalf.

Thank you for bringing this to our attention. We confirm that the funders, including the Saudi Arabian Ministry of Education and King Abdulaziz University, DSR in Jeddah, had no role in the study design, data collection and analysis, decision to publish, or preparation of the manuscript. We will include this amended Role of Funder statement in our cover letter as requested. If any further adjustments are needed, please let us know.

Please provide a complete Data Availability Statement in the submission form, ensuring you include all necessary access information or a reason for why you are unable to make your data freely accessible. If your research concerns only data provided within your submission, please write "All data are in the manuscript and/or supporting information files" as your Data Availability Statement

We confirm that all data relevant to our study are included in the manuscript. We will provide a complete Data Availability Statement in the submission form accordingly. If you require any further information or assistance, please let us know.

Thank you for stating the following in the Acknowledgments Section of your manuscript:

“Grant number IFPIP:1281-140-1443 from the Instructional Improvement Fund supported this study. The authors would like to express their appreciation to the Saudi Arabian Moe and King Abdulaziz University, DSR in Jeddah for their technical and financial aid”

We note that you have provided funding information that is currently declared in your Funding Statement. However, funding information should not appear in the Acknowledgments section or other areas of your manuscript. We will only publish funding information present in the Funding Statement section of the online submission form.

Please remove any funding-related text from the manuscript and let us know how you would like to update your Funding Statement. Currently, your Funding Statement reads as follows:

“Grant number IFPIP:1199-140-1443- 260449 from the Instructional Improvement Fund supported this study.  Saudi Arabian Moe and King Abdulaziz University, DSR in Jeddah.”

Please include your amended statements within your cover letter; we will change the online submission form on your behalf.

Thank you for bringing this to our attention. We apologize for the oversight regarding the inclusion of funding information in the Acknowledgments section of our manuscript. We will promptly remove any funding-related text from the manuscript.

Regarding the Funding Statement, please amend it to the following:

The authors would like to express their appreciation to the Saudi Arabian Ministry of Education and King Abdulaziz University, DSR in Jeddah for their technical and financial aid."

We will include this amended Funding Statement in our cover letter as requested. If any further adjustments are needed, please let us know.

Reviewers' comments:

Reviewer's Responses to Questions

**Comments to the Author**

Please use the space provided to explain your answers to the questions above. You may also include additional comments for the author, including concerns about dual publication, research ethics, or publication ethics. (Please upload your review as an attachment if it exceeds 20,000 characters)

Reviewer #1: The manuscript entitled “In-Vivo and In-Vitro Toxicity Evaluation of 2,3-Dimethylquinoxaline: an Antimicrobial Found in a Traditional Herbal Medicine” is worth publishing after applying some minor corrections. My comments are as following:
1. In the names and affiliations of the authors there are some mistakes that need to be corrected:
• The number of the author “Abdelbagi Alfadil13” as superscript is 13 please correct,
• There should be a comma between the second and the first author,
• The comma after the number of the 4th author “Huda Alkreathy2,” should be removed,
• There are dots after the numbers of the 5th and 6th authors which should be removed, also there should be a comma between their names.

Abdelbagi Alfadil^1,3^^*^, Hamoud Alsamhan^2^, Ahmed Ali^2^, Huda Alkreathy^2,^

, Mohammad W. Alrabia^1^, Asif fatani^1^, Karem A Ibrahem^1^

• The font size and the and the font type should be uniform in the 1st page

Abdelbagi Alfadil^1,3*^, Hamoud Alsamhan^2^, Ahmed Ali^2^, Huda Alkreathy^2,^

, Mohammad W. Alrabia^1^, Asif fatani^1^, Karem A Ibrahem^1^

1- Department of Clinical Microbiology and Immunology, Faculty of Medicine, King Abdulaziz University, P.O. Box 80205, Jeddah 21589, Saudi Arabia. ([aegmusa@kau.edu.sa](mailto:aegmusa@kau.edu.sa)) ([kaibrahem@kau.edu.sa](mailto:kaibrahem@kau.edu.sa)) ([mwalrabia@kau.edu.sa](mailto:mwalrabia@kau.edu.sa)) ([afatani@kau.edu.sa](mailto:afatani@kau.edu.sa))

2- Department of Clinical Pharmacology, Faculty of Medicine, King Abdulaziz University, Jeddah 21589, Saudi Arabia. ([Samhanh@gmail.com](mailto:Samhanh@gmail.com)) ([asali@kau.edu.sa](mailto:asali@kau.edu.sa)) ([halkreathy@kau.edu.sa](mailto:halkreathy@kau.edu.sa))

3- Centre of Research Excellence for Drug Research and Pharmaceutical Industries, King Abdulaziz University, Jeddah, Saudi Arabia.

***Corresponding author**

Abdelbagi Alfadil Department of Clinical Microbiology and Immunology, Faculty of Medicine, King Abdulaziz University, Jeddah, Saudi Arabia, , P.O. Box 80205, Jeddah 21589, Saudi Arabia. Tel: +96612 6952000 Ext:21062 .

Email : [aegmusa@kau.edu.sa](mailto:aegmusa@kau.edu.sa).

2. Authors should start with general statements about the relevant subject in the introduction part,

Natural compounds derived from plants have long been recognized for their diverse array of bioactive properties, which are integral to their medicinal significance (Gyawali and Ibrahim, 2014). These compounds, often referred to as phytochemicals, exhibit a wide range of biological activities that can influence human health in various ways. From antimicrobial and anti-inflammatory effects to antioxidant and anticancer properties, these bioactive molecules hold immense potential for therapeutic applications (Selek et al., 2018; El Newahie et al., 2019; Raphoko et al., 2021; Meka and Chintakunta, 2023)

However, despite the promising pharmacological activities exhibited by natural compounds, their safety profile remains a critical concern that warrants thorough investigation . Establishing the safety of these compounds is paramount before considering their utilization in pharmaceutical products or clinical interventions (Barba-Ostria et al., 2022). This is due to the inherent variability and complexity of natural compounds, which can pose challenges in predicting their potential adverse effects and interactions within biological systems (Barba-Ostria et al., 2022).

Conducting comprehensive studies to elucidate the safety profile of natural compounds involves a multidisciplinary approach that encompasses various aspects of toxicology, pharmacology, and molecular biology (Khan, 2018). These investigations aim to assess the potential toxicity, pharmacokinetics, and organ-specific effects of natural compounds through a series of *in vitro* and *in vivo* experiments (Wang et al., 2008).

Furthermore, understanding the mechanisms underlying the biological activities of natural compounds is essential for discerning their potential therapeutic benefits and adverse effects (Qneibi et al., 2024). By elucidating the molecular pathways and targets involved, researchers can gain insights into the safety and efficacy of these compounds, thereby facilitating their translation into clinical applications (Hao et al., 2014).

4. The title “2.9 Oral toxicity study with repeated doses in mice (28 days )” should be written with bold

Thank you for the feedback. We will ensure that the title "2.9 Oral toxicity study with repeated doses in mice (28 days)" is written in bold font as suggested.

## 2.9 Oral toxicity study with repeated doses in mice (28 days)

5. Authors should write a more detailed conclusion

DMQ demonstrates broad-spectrum antimicrobial properties with potential therapeutic applications. This study evaluated its toxicity through *in vitro* and *in vivo* experiments, revealing no significant adverse effects at ≤100 µM concentrations *in vitro* and a high median lethal dose (>2000 mg/kg) in animal studies. However, mild histological changes were observed at high doses. Overall, DMQ shows an acceptable safety profile in rodents, supporting its potential as a therapeutic agent. These also support the continued development of DMQ and its derivatives to further progress into the antimicrobial pipeline. Future toxicity studies are needed in non-rodent species to enhance the generalizability of these findings across species.

6. Please add the following related references in the introduction part
• Yener İsmail, Yilmaz Mustafa Abdullah, Tokul Ölmez Özge, Akdeniz Mehmet, Tekin Fethullah, Haşimi Nesrin, Alkan Mehmet Hüseyin, Öztürk Mehmet, Ertaş Abdulselam (2020). A Detailed Biological and Chemical Investigation of Sixteen Achillea Species’ Essential Oils via Chemometric Approach. CHEMISTRY & BIODIVERSITY, 17(3) e1900484, <https://doi.org/10.1002/cbdv.201900484>.

It has been shown that the chemical composition and biological effects of essential oils from sixteen Achillea species in herbal dietary supplements. It found moderate antioxidant, antimicrobial, and enzyme inhibition effects across most samples. Chemometric analysis revealed distinct variations among species, with A. nobilis subsp. neilreichii showing unique characteristics (Yener et al., 2020).

• Selek Şehabettin, Koyuncu İsmail, Çağlar Hifa Gülru, Bektaş İbrahim, Yilmaz Mustafa Abdullah, Gönel Ataman, Akyüz Enes (2018). The evaluation of antioxidant and anticancer effects of Lepidium Sativum Subsp Spinescens L. methanol extract on cancer cells. Cellular and Molecular Biology, 64(3), 72-80., <https://doi.org/10.14715/cmb/2018.64.3.12>.

A study investigates the antioxidant content and activity of Lepidium sativum Subsp spinescens L. methanol extract on cancer cells. The extract exhibited high phenolic and flavonoid content, significant antioxidant activity, and concentration-dependent cytotoxic effects on colon and endometrium cancer cells, suggesting its potential as a natural antioxidant source with possible anticancer properties that warrant further molecular studies (Selek et al., 2018).

• Mehmet Akdeniz, Ismail Yener, Mustafa Abdullah Yilmaz, Sevgi Irtegun Kandemir, Fethullah Tekin, Abdulselam Ertas (2021). A potential species for cosmetic and pharmaceutical industries: Insight to chemical and biological investigation of naturally grown and cultivated Salvia multicaulis Vahl. Industrial Crops & Products, 168, 113566, <https://doi.org/10.1016/j.indcrop.2021.113566>.

Another study showed that the biological and chemical properties of essential oils and ethanol extracts from naturally grown and cultivated Salvia multicaulis Vahl. The analysis reveals high enzyme and antioxidant activities in the extracts, particularly in leaf parts, with significant cytotoxic effects observed in various cancer cell lines. Major components in the essential oils differ between natural and cultivated specimens, while both types show richness in β-Sitosterol and rosmarinic acid, suggesting potential applications in food supplements and pharmaceuticals. (Akdeniz et al., 2021).

• Mehmet Akdeniz, Ismail Yener, Abdulselam Ertas, Mehmet Firat, Baris Resitoglu, Nesrin Hasimi, Sevgi Irtegun Kandemir, Mustafa Abdullah Yilmaz, Asli Barla Demirkoz, Ufuk Kolak and Sevil Oksuz (2021). Biological and Chemical Comparison of Natural and Cultivated Samples of Satureja macrantha C.A.Mey. Records of Natural Products, 15 (6), 568-584, <http://doi.org/10.25135/rnp.237.21.02.1957>.

Akdeniz et al showed that the essential oils and extracts of both naturally grown and cultivated Satureja macrantha, analyzing their chemical composition and biological effects. The cultivated sample exhibited higher phenolic content and antioxidant capacity, with differences in major components impacting biological properties, likely influenced by environmental factors (Akdeniz et al., 2021).

34. Ebubekir Izol, Hamdi Temel, Mustafa Abdullah Yilmaz, Ismail Yener, Ozge Tokul Olmez, Erhan Kaplaner, Mehmet Fırat, Nesrin Hasimi, Mehmet Ozturk, Abdulselam Ertas (2021). A Detailed Chemical and Biological Investigation of Twelve Allium Species from Eastern Anatolia with Chemometric Studies. Chemistry and Biodiversity, 18 (1), e2000560, <https://doi.org/10.1002/cbdv.202000560>.

Ethanol extracts of aerial parts and roots from 12 Allium species were analyzed for phenolic content and tested for antioxidant, anticholinesterase, cytotoxic, and antimicrobial activities. Aerial parts exhibited better antioxidant activity, and some species showed high cytotoxic and antimicrobial effects, suggesting potential pharmaceutical and food industry applications that warrant further investigation. (Izol et al., 2021).

Reviewer #2: The document submitted for our consideration is a scientific work of significance, providing information on the toxicological profile of 2,3-dimethylquinoxaline (DMQ), a broad-spectrum antimicrobial derived from plants.

Abstract
In vitro and in vivo remain a group of words which are necessarily italicised.

The terms "in vitro" and "in vivo" continue to be italicized in scientific literature.

*in vivo* *in vitro*

Introduction
Chromolaenaodorata is in two words Chromolaena odorata

*Chromolaenaod orata*
Please update the references in the second paragraph of the introduction. The references used are too old.

Certainly, I will update the references in the second paragraph of the introduction with more recent sources while ensuring that the original paper is retained. (Izol et al., 2021). (Akdeniz et al., 2021).

Material and methods
You were right to specify in this study that the mice used were non-pregnant females. Are they nulliparous or multiparous? These are parameters to be taken into account for the tests.
This choice must be justified

Thank you for bringing up this important point. The mice used in this study were non-pregnant nulliparous females. This choice was made to minimize potential confounding variables related to reproductive history and hormonal fluctuations, ensuring more accurate and consistent test results.
Laboratory animals, particularly females, are more sensitive than males and when females are pregnant or nulliparous, the tests may be influenced by these physiological characteristics. Here are some of the reasons why it is important to specify the physiological characteristics of the animals tested

Reference list

Akdeniz, M., Yener, I., Yilmaz, M. A., Kandemir, S. I., Tekin, F., & Ertas, A. (2021) ‘A potential species for cosmetic and pharmaceutical industries : Insight to chemical and biological investigation of naturally grown and cultivated Salvia multicaulis Vahl.’

Akdeniz, M., Yener, I., Ertas, A., Firat, M., Resitoglu, B., Hasimi, N., Irtegun Kandemir, S., Yilmaz, M. A., Barla Demirkoz, A., Kolak, U. and Oksuz, S. (2021) ‘Biological and chemical comparison of natural and cultivated samples of satureja macrantha c.A.mey.’ *Records of Natural Products*, 15(6) pp. 568–584.

Barba-Ostria, C., Carrera-Pacheco, S. E., Gonzalez-Pastor, R., Heredia-Moya, J., Mayorga-Ramos, A., Rodríguez-Pólit, C., Zúñiga-Miranda, J., Arias-Almeida, B. and Guamán, L. P. (2022) ‘Evaluation of Biological Activity of Natural Compounds: Current Trends and Methods.’ *Molecules*, 27(14) pp. 1–35.

Gyawali, R. and Ibrahim, S. A. (2014) ‘Natural products as antimicrobial agents.’ *Food Control*, 46 pp. 412–429.

Hao, H., Zheng, X. and Wang, G. (2014) ‘Insights into drug discovery from natural medicines using reverse pharmacokinetics.’ *Trends in Pharmacological Sciences*. Elsevier Ltd, 35(4) pp. 168–177.

Izol, E., Temel, H., Yilmaz, M. A., Yener, I., Olmez, O. T., Kaplaner, E., Fırat, M., Hasimi, N., Ozturk, M. and Ertas, A. (2021) ‘A Detailed Chemical and Biological Investigation of Twelve Allium Species from Eastern Anatolia with Chemometric Studies.’ *Chemistry and Biodiversity*, 18(1) p. 12.

Khan, R. A. (2018) ‘Natural products chemistry: The emerging trends and prospective goals.’ *Saudi Pharmaceutical Journal*. King Saud University, 26(5) pp. 739–753.

Meka, G. and Chintakunta, R. (2023) ‘Analgesic and anti-inflammatory activity of quinoxaline derivatives: Design synthesis and characterization.’ *Results in Chemistry*. Elsevier B.V., 5(October 2022) p. 100783.

El Newahie, A. M. S., Nissan, Y. M., Ismail, N. S. M., Abou El Ella, D. A., Khojah, S. M. and Abouzid, K. A. M. (2019) ‘Design and synthesis of new quinoxaline derivatives as anticancer agents and apoptotic inducers.’ *Molecules*, 24(6).

Qneibi, M., Bdir, S., Maayeh, C., Bdair, M., Sandouka, D., Basit, D. and Hallak, M. (2024) *A Comprehensive Review of Essential Oils and Their Pharmacological Activities in Neurological Disorders: Exploring Neuroprotective Potential*. *Neurochemical Research*.

Raphoko, L. A., Lekgau, K., Lebepe, C. M., Leboho, T. C., Matsebatlela, T. M. and Nxumalo, W. (2021) ‘Synthesis of novel quinoxaline-alkynyl derivatives and their anti-Mycobacterium tuberculosis activity.’ *Bioorganic and Medicinal Chemistry Letters*, 35 p. 127784.

Selek, S., Koyuncu, I., Caglar, H. G., Bektas, I., Yilmaz, M. A., Gonel, A. and Akyuz, E. (2018) ‘The evaluation of antioxidant and anticancer effects of Lepidium Sativum Subsp Spinescens L. methanol extract on cancer cells.’ *Cellular and Molecular Biology*, 6(3) pp. 72–80.

Wang, W., Wang, H., Rayburn, E. R., Zhao, Y., Hill, D. L. and Zhang, R. (2008) ‘20(S)-25-methoxyl-dammarane-3β, 12β, 20-triol, a novel natural product for prostate cancer therapy: Activity in vitro and in vivo and mechanisms of action.’ *British Journal of Cancer*, 98(4) pp. 792–802.

Yener, I., Yilmaz, M. A., Olmez, O. T., Akdeniz, M., Tekin, F., Hasimi, N., Alkan, M. H., Ozturk, M. and Ertas, A. (2020) ‘A Detailed Biological and Chemical Investigation of Sixteen Achillea Species’ Essential Oils via Chemometric Approach.’ *Chemistry and Biodiversity*, 17(3).
