# Supplementary material for: In-vivo and in-vitro toxicity evaluation of 2,3-dimethylquinoxaline: An antimicrobial found in a traditional herbal medicine
Source: PLoS One. 2024 Aug 20;19(8):e0300079. doi: 10.1371/journal.pone.0300079 (PMC11335103; doi:10.1371/journal.pone.0300079)
Supplement: Attachment — Submitted filename: REVIEWER COMMENTS.docx [file pone.0300079.s001.docx]

**REVIEWER COMMENTS**

The manuscript entitled “In-Vivo and In-Vitro Toxicity Evaluation of 2,3-Dimethylquinoxaline: an Antimicrobial Found in a Traditional Herbal Medicine” is worth publishing after applying some minor corrections. My comments are as following:

1. In the names and affiliations of the authors there are some mistakes that need to be corrected:

- The number of the author “Abdelbagi Alfadil^13^” as superscript is 13 please correct,
- There should be a comma between the second and the first author,
- The comma after the number of the 4th author “Huda Alkreathy^2,^” should be removed,
- There are dots after the numbers of the 5th and 6th authors which should be removed, also there should be a comma between their names.
- The font size and the and the font type should be uniform in the 1st page

2. Authors should start with general statements about the relevant subject in the introduction part,
3. Authors should extend the introduction part with explanatory and literary comments.
4. The title “2.9 Oral toxicity study with repeated doses in mice (28 days )” should be written with bold
5. Authors should write a more detailed conclusion
6. Please add the following related references in the introduction part

- Yener İsmail, Yilmaz Mustafa Abdullah, Tokul Ölmez Özge, Akdeniz Mehmet, Tekin Fethullah, Haşimi Nesrin, Alkan Mehmet Hüseyin, Öztürk Mehmet, Ertaş Abdulselam (2020). A Detailed Biological and Chemical Investigation of Sixteen *Achillea* Species’ Essential Oils via Chemometric Approach. CHEMISTRY & BIODIVERSITY, 17(3) e1900484, <https://doi.org/10.1002/cbdv.201900484>.
- Selek Şehabettin, Koyuncu İsmail, Çağlar Hifa Gülru, Bektaş İbrahim, Yilmaz Mustafa Abdullah, Gönel Ataman, Akyüz Enes (2018). The evaluation of antioxidant and anticancer effects of *Lepidium Sativum* Subsp *Spinescens* L. methanol extract on cancer cells. Cellular and Molecular Biology, 64(3), 72-80., <https://doi.org/10.14715/cmb/2018.64.3.12>.
- Mehmet Akdeniz, Ismail Yener, Mustafa Abdullah Yilmaz, Sevgi Irtegun Kandemir, Fethullah Tekin, Abdulselam Ertas (2021). A potential species for cosmetic and pharmaceutical industries: Insight to chemical and biological investigation of naturally grown and cultivated *Salvia* *multicaulis* Vahl. Industrial Crops & Products, 168, 113566, <https://doi.org/10.1016/j.indcrop.2021.113566>.
- Mehmet Akdeniz, Ismail Yener, Abdulselam Ertas, Mehmet Firat, Baris Resitoglu, Nesrin Hasimi, Sevgi Irtegun Kandemir, Mustafa Abdullah Yilmaz, Asli Barla Demirkoz, Ufuk Kolak and Sevil Oksuz (2021). Biological and Chemical Comparison of Natural and Cultivated Samples of *Satureja* *macrantha* C.A.Mey. Records of Natural Products, 15 (6), 568-584, <http://doi.org/10.25135/rnp.237.21.02.1957>.
- 34.	Ebubekir Izol, Hamdi Temel, Mustafa Abdullah Yilmaz, Ismail Yener, Ozge Tokul Olmez, Erhan Kaplaner, Mehmet Fırat, Nesrin Hasimi, Mehmet Ozturk, Abdulselam Ertas (2021). A Detailed Chemical and Biological Investigation of Twelve *Allium* Species from Eastern Anatolia with Chemometric Studies. Chemistry and Biodiversity, 18 (1), e2000560, <https://doi.org/10.1002/cbdv.202000560>.
